# Experimental and Numerical Investigations on High Performance SFRC: Cyclic Tensile Loading and Fatigue

**DOI:** 10.3390/ma14247593

**Published:** 2021-12-10

**Authors:** Niklas Schäfer, Vladislav Gudžulić, Rolf Breitenbücher, Günther Meschke

**Affiliations:** 1 Institute for Building Materials Technology, Ruhr-University Bochum, 44801 Bochum, Germany; rolf.breitenbuecher@ruhr-uni-bochum.de; 2 Institute for Structural Mechanics, Ruhr-University Bochum, 44801 Bochum, Germany; vladislav.gudzulic@ruhr-uni-bochum.de (V.G.); guenther.meschke@ruhr-uni-bochum.de (G.M.)

**Keywords:** SFRC, fatigue, tensile stress, controlled crack opening test, cohesive zone model, discrete fibers, crack closure model

## Abstract

In the present study, the capability of high-strength short steel fibers to control the degradation in high-performance concrete was experimentally examined and numerically simulated. To this end, notched prismatic high-performance concrete specimens with (HPSFRC) and without (HPC) short steel fibers were subjected to static and cyclic tensile tests up to 100,000 cycles. The cyclic tests showed that the rate of strain increase was lower for HPSFRC specimens and that the strain stagnated after around 10,000 cycles, which was not the case with HPC specimens. The microscopic examinations showed that in HPSFRC, a larger number of microcracks developed, but they had a smaller total surface area than the microcracks in the HPC. To further investigate the influence of fibers on the behavior of HPSFRC in the cracked state, displacement-controlled crack opening tests, as well as numerical simulations thereof, were carried out. Experiments have shown, and the numerical simulations have confirmed, that the inclusion of short steel fibers did not significantly affect the ultimate strength; however, it notably increased the post-cracking ductility of the material. Finally, the unloading/reloading behavior was examined, and it was observed that the unloading stiffness was stable even for significant crack openings; however, the hysteresis loops due to unloading/reloading were very small.

## 1. Introduction

Increasing requirements in terms of slender design, material savings, and increased durability make the use of high-performance building materials indispensable. Slender constructions, such as bridge girders, onshore and offshore installations [1] which are affected by time-variant simultaneous loading of for example traffic, wind, and waves [2], are vulnerable to fatigue loads that can ultimately lead to a failure of the supporting structure [3]. Slender constructions are generally more sensitive to variable external loads, which result in vibration [4]. This may cause material fatigue, leading to damage or even failure before the maximum static strength is achieved [5]. In concrete, there are three phases of damage evolution that finally lead to a fatigue failure. Initially, a large number of microcracks is created (phase I). After this, a further steady formation of microcracks occurs (phase II), which is significantly slower than during phase I. The formation of a coherent crack network ultimately leads to the final failure (phase III) [4]. Compared to normal strength concrete, the phase I of damage evolution is shorter, and generally, the strain is higher in high-strength concrete [6,7,8].

The addition of steel fibers typically improves the properties of high-performance concrete under tensile stress. The effectiveness of fibers depends on different factors, for example, fiber geometry, fiber slenderness (length to diameter ratio), and fiber amount [9,10,11,12,13]. Fibers used in concrete construction can transfer tensile forces at least partially over the cracks after their formation and consequently improve the ductility of the comparatively brittle concrete [14]. The influence on the crack formation is of particular importance. It is characterized by the interaction behavior of individual fibers with the surrounding matrix. By default, macrofibers (*Ø* > 300 μm) are used in the construction industry. A major disadvantage is that they are activated and transmit tensile stresses only across the cracks with a width greater than 50 μm. Microfibers with a diameter smaller than 100 μm should be used to effectively bridge microcracks with crack widths much smaller than 50 μm [15]. This is because in the case of microfibers with a diameter larger than 300 µm, the intact concrete ligaments still transfer the tensile stresses across the crack, and the fibers are not activated because they are too thick compared to the crack width. In order to transfer the tensile stresses across the cracks with widths smaller than 50 µm, microfibers with smaller diameters are necessary; see also [16]. Microfibers can transfer the stresses across the cracks and thus inhibit the growth and propagation of the cracks, which enables the SFRC to be extensively and successfully used for crack control [17]. The bond strength between the matrix and the fiber is an important material parameter that controls the activation of fibers, i.e., the transfer of tensile stresses from concrete to fibers. If the specific fiber surface or the stress-transferring lateral area increases, larger pullout resistance is possible. The bond properties that characterize the composite behavior of fibers and cementitious matrix are typically determined by pullout tests of individual fibers. If smooth, straight fibers without hooked ends are used, the bond strength can arise initially through adhesive bonding and friction between the matrix and fiber surface. The frictional sliding only occurs when the adhesive bond strength has been exceeded. The bond strength can be increased by the mechanical interlock of fiber and matrix. A profiled fiber surface, a non-straight fiber geometry (e.g., wavy fibers), and hooked-end fibers are commonly used [18,19,20]. If a comparison is made with special regard to microcracking, fiber reinforced concrete shows a much more finely distributed crack pattern with smaller crack spacings and crack widths than conventionally reinforced concrete [19]. Recent experimental-numerical campaigns have elucidated some of the mechanisms that contribute to the resilience of fiber-reinforced cementitious composites under cyclic loading (see [21,22]).

Numerous attempts have been made to develop models that describe the homogenized behavior of plain and fiber-reinforced concrete entirely, under monotonic (e.g., [23,24]) as well as under cyclic loading (e.g., [25,26,27]). However, a definitive consensus has yet to be reached. In the finite element simulations, explicit modeling of discrete fibers has always been computationally challenging, and in [28], a novel method for simulation of a large number of fibers has been presented. Due to the substantial computational complexity of elaborate numerical models, the influence of cyclic loading on the fatigue lifetime of concrete structures is in practical design typically estimated using relatively simple qualitative checks and empirical damage accumulation rules [29]. Slightly more involved phenomenological models are based on the accumulation of certain internal variables such as plastic strain or dissipated energy. A comparison concerning the ability to reproduce Wöhler curves, hysteretic loops in unloading and reloading, and the effects of loading sequence of relevant phenomenological model for fatigue in compression is given in [30]. Further, for fatigue crack growth in concrete and other quasibrittle materials, Paris law adjusted for accounting the size effect is often used [31]. Even though empirical and phenomenological models provide a way to estimate the resistance of a material to fatigue crack initiation and growth, they do not provide a mechanistic description of how microstructure (~10^−6^ m–10^−3^ m [32]) around the crack tip affects crack growth and does not fully explain the resistance of the material to fatigue [33]. The mechanisms occurring near crack tip are termed as intrinsic if they promote the crack growth (e.g., microcrack coalescence) and extrinsic if they impede it (e.g., crack closure and crack face bridging [34]). Since a significant portion of the fatigue lifetime of concrete is spent under low loading, the crack tip opening displacements are typically comparable to the characteristic size of the microstructure and much smaller than the typical aggregate size, so they are very much influenced by the microscopic and mesoscopic features such as pores and aggregates. However, such mechanisms substantially affect the global response, evidenced in the formation of hysteretic loops. For example, the crack closure mechanism explains how fatigue cracks can remain closed even under tensile loading [35]. Additionally, post-mortem analyses of failed structural components often reveal certain fracture surface features, which can be related to the cause of failure only if the fundamental mechanisms are well understood [33].

In the present work, the influence of the addition of short steel fibers to high-performance concrete on mechanical and durability characteristics was analyzed through a set of static and cyclic tensile tests up to 100,000 cycles on notched prismatic specimens. Additionally, the post-cracking behavior of short steel fiber reinforced concrete was investigated by performing and numerically simulating the controlled crack opening test. In the numerical model, every fiber was resolved using Bernoulli beam finite elements, which enabled simulations with arbitrary spatial distribution and orientation of fibers. Finally, we investigated the unloading/reloading behavior experimentally and numerically utilizing a crack closure model that accounts for the formation of hysteresis loops due to internal frictional mechanisms.

## 2. Experimental Investigations

The experimental investigations consisted of three parts:

1. Pull-out tests to assess the bond characteristics of individual steel fibers embedded in a high-strength mortar.
2. The fatigue behavior of high-strength concrete with and without high-strength steel fibers under cyclic tension.
3. The post-peak ductility and unloading/reloading behavior test performed on short steel fiber reinforced high-performance concrete (HPSFRC), assessed through the crack opening test with the predefined displacement-controlled loading scenario.

Single fiber pull-out tests were used to investigate the interfacial bond strength, toughness, and load transfer between high-strength mortar and high-strength steel microfibers. These parameters are necessary to calibrate the numerical model, described in detail in Section 5. Due to the small fiber dimensions, we used a mortar composition (HPC-02) derived from the high-strength concrete composition (HPC-08) in pull-out tests. To investigate the fatigue behavior of high-strength concrete with and without high-strength steel microfibers, a series of cyclic tensile fatigue load experiments was carried out. These tests examined the strains vs. the number of cycles (S/N curves), the drop in stiffness, and the crack closure and reopening mechanics under changing loads.

## 3. Materials and Methods

The material composition of the investigated specimens is shown in Table 1. For all mixtures, Portland cement CEM I 52.5 R was used. Fine quartz sand, mineral sand, and basalt with a maximum grain size of 8 mm were used as aggregates. Additionally, a superplasticizer and a stabilizer were added. From the basic composition (HPC-08), where “08” represents the maximum grain size of 8 mm, a high-strength mortar composition (HPC-02) was derived by eliminating all aggregates with a grain size above 2 mm. The water-cement value (*w*/*c* value) of 0.35 was kept constant for all mixtures. For comparable fresh properties, the amount of superplasticizer was adjusted for the HPSFRC (HPC-08-SF). Bekaert short steel fibers with a diameter of 0.160 mm and a length of 6 mm (Dramix OL 6/0.16) were added to the mixture. According to our previous experience with fresh concrete mixes, fibers only in an amount of about 70 to 100 kg/m^3^ can be mixed reliably, so we chose a fiber dosage of 1% by volume (see Table 1). For the characterization of bond properties between mortar and fibers, single fiber pull-out tests were devised. Since the Dramix OL 6/0.16 fiber was too short for such tests, we decided to use a single long fiber in the pull-out tests. Due to manufacturing reasons, we could not obtain these fibers from the same manufacturer as the short fibers. However, a fiber with comparable dimensions and properties was chosen for the single fiber pull-out tests (see Table 2). The material properties of the hardened concrete are given in Table 3.

## 4. Test Methods

### 4.1. Pull-Out Tests

**Figure 1:**
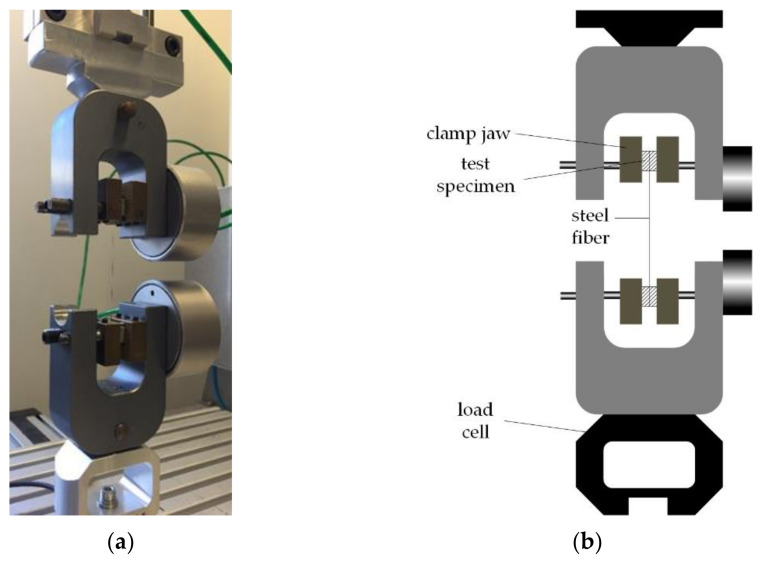
Test setup for pull-out tests: (**a**) Physical; (**b**) Schematic [15]. Reproduced from the Proceedings of the fib Symposium 2019: Concrete Innovations in Materials, Design and Structures, paper “Pull-Out tests of carbon and high-strength steel microfibres in a cement-based matrix”, page 1893 -” Figure 5: Tests set-up for pull-out tests, left: for carbon fibre, middle and right: for steel fibre” with permission from the International Federation for Structural Concrete (fib).

The pull-out tests were carried out on a tensile test machine (maximum load: 160 N), shown in Figure 1a. The test was displacement-controlled with a constant displacement rate of 0.1 mm/s. In the tests, the specimen consisted of one long fiber bound on both sides in a high-strength mortar (HPC-02), as shown in Figure 1b. The test specimens were fixed in the testing machine by clamping jaws and held at a constant contact pressure of 6 bar, ensuring the same clamping force for every test. The load application was parallel to the fiber, and the fiber pull-out force was measured by a load cell with a maximum capacity of 50 N.

### 4.2. Static and Cyclic Tensile Tests

**Figure 2:**
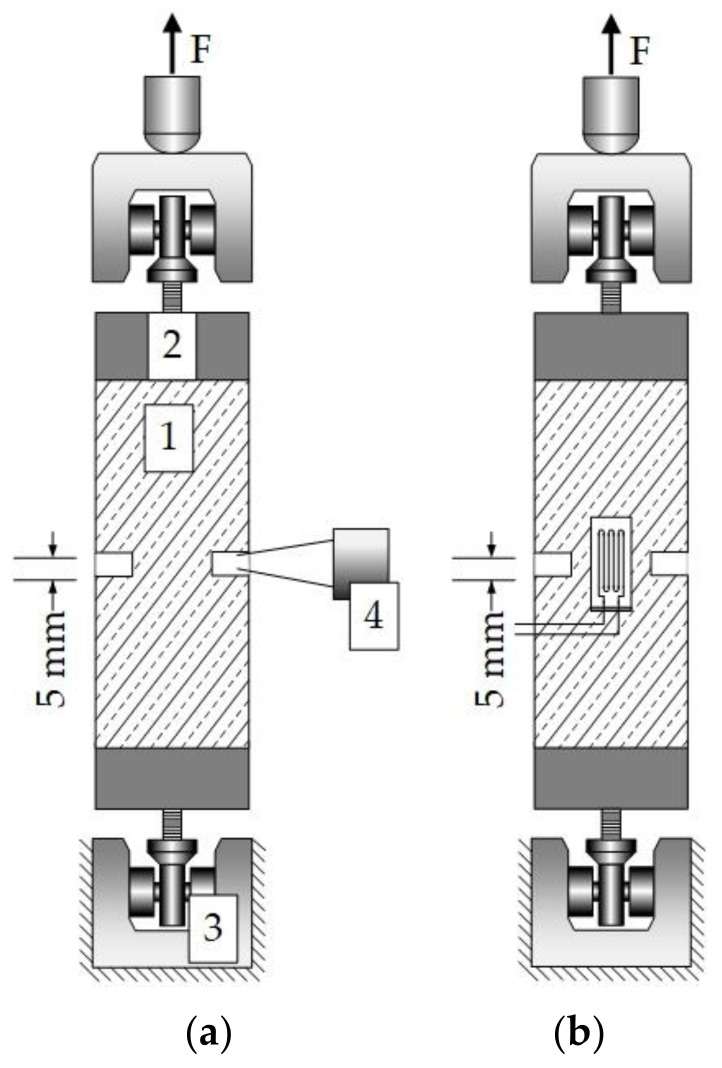
Test setups: (**a**) static tensile test setup including the specimen 1, the two adhered test stamps (bottom/top) 2, the lower/upper clamping in the machine 3, and the clip-on-gage 4; (**b**) cyclic tensile test setup with strain gauges.

The static tensile tests were performed to determine the maximum static tensile strength. We used prismatic (40 mm × 40 mm × 160 mm) specimens with two notches (5 mm in width, 10 mm in depth) cut in the middle of the specimens for static and cyclic investigations. The notches, introduced on two sides, define the weakest section, subsequently the breaking point. Based on strength values obtained from the static tensile test, we defined the upper and lower fatigue limit stresses as particular strength percentages. The static tensile tests, schematically shown in Figure 2a, were carried out in an electro-mechanical 250 kN testing machine, while the cyclic tensile tests, schematically shown in Figure 2b, were carried out in a servo-hydraulic 100 kN tensile testing machine. Both testing machines are from the manufacturer Schenck and the maximum forces were detected by means of loads cells with a capacity of 100 kN. In displacement-controlled experiments, the test specimens were loaded very slowly, with a constant displacement rate of 0.1 mm/min until failure of the specimen occurred. In force-controlled fatigue tests, the test specimens were subjected to tensile stresses according to the sinusoidal load functions with an upper load level of 70% of the maximum static tensile strength *f*_ct, max_ and a lower level of 35% *f*_ct, max_, see Table 4.

The cyclic loading was stopped after reaching up to 100,000 cycles. The displacement in the static tensile tests was measured by a clip-on-gage (Figure 2a), whereas in the cyclic tensile tests, the displacements in the predefined breaking points (area of the notch) were measured using strain gages (Figure 2b).

**Figure 3:**
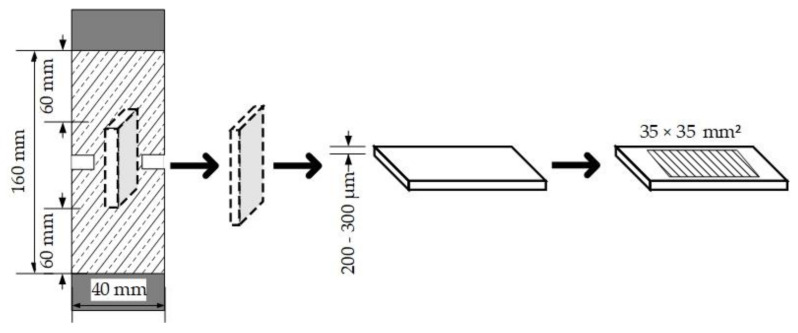
Preparation of thick sections from the middle region (notch region) of the test specimen.

After 0, 1, 100, 1000 and 100,000 load cycles, one test specimen of the three tested specimens in each series was chosen, from which the partial samples for microscopic examinations (amount, length, width, and position of the microcracks) were prepared as thick sections using colored resin. Thick sections refer to the preparation of slices of a thickness of 200–300 µm, see Figure 3. Therefore, a slice of the test specimen in the loading direction (notch region) was resected and impregnated with resin under vacuum and then polished. This process was repeated until the thickness of 200–300 µm was reached. We performed the impregnation under vacuum so that the microcracks would not be filled with dust from polishing since they were already filled with the resin. On this thick section an examination field of 35 × 35 mm^2^ with a raster of 7 × 7 cells was plotted. The thick sections were prepared after the specimens were subjected to load cycles, so it was possible to inspect the crack evolution in HPC-08 and HPC-08-SF.

### 4.3. Crack Opening Tests

**Figure 4:**
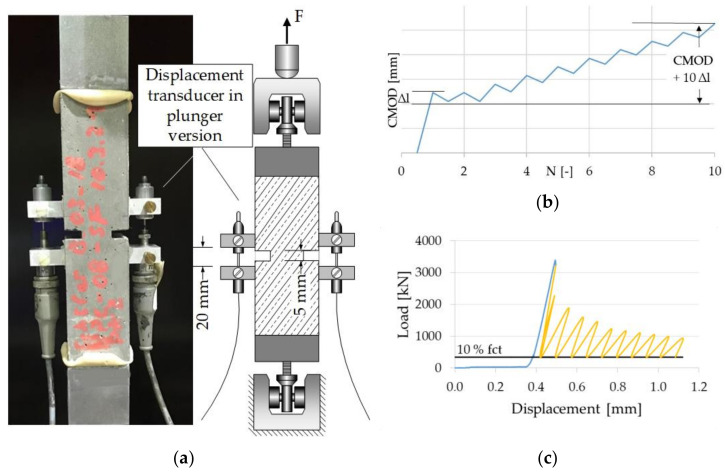
Experiment on notched prismatic specimen: (**a**) Tensile test setup: real and schematic; (**b**) test regime of crack opening controlled tests; (**c**) example of the loading procedure for the crack opening test.

To investigate the crack closing and re-opening mechanisms, crack opening tests were performed as follows. Notched prismatic test specimens with the same dimensions as in the case of static and cyclic investigations (see Section 4.2) were used. For these investigations, an electro-mechanical 250 kN testing machine, as shown in Figure 4a, was utilized. This time, however, displacement transducers in the plunger version (HBM W1ELA/0) were used to measure the strain. The displacement transducers in the plunger version have a maximum measuring range of 20 mm and were attached to the notch. The sample was subjected to a monotonic tensile loading until the first crack appeared. The sample was then unloaded to 10% of the tensile strength, and the difference in strain (Δl) was measured. The displacement was then prescribed on the top end of the specimen until the value of the strain measured at the notch area reached Δl, followed by unloading to 10% of the tensile strength (see Figure 4b). Subsequently, the procedure of crack opening controlled loading until the crack width of CMOD + 10 Δl was reached, and subsequent force-controlled relieving of the sample was repeated, as illustrated in Figure 4c.

## 5. Numerical Model

The notched prismatic fiber-reinforced concrete specimens undergo controlled cyclic loading and unloading, which is assumed to be a quasi-static process in the numerical simulations. The equilibrium equation reads:

(1) divσ+ρb=0,

with σ being the Cauchy stress tensor, ρ the mass density, and b the specific body force vector. Equation (1) is recast into the weak form and discretized and solved employing the finite element method. The concrete cracking is modeled by the zero-thickness interface elements equipped with the traction-separation law, explained in Section 5.1. Individual fibers are resolved explicitly using Bernoulli beam finite elements and bond elements that model the interaction between concrete and fibers, detailed in Section 5.2. The overview of the complete model and the boundary conditions are given in Section 5.3.

### 5.1. Cohesive Zone Model for Concrete Cracking

**Figure 5:**
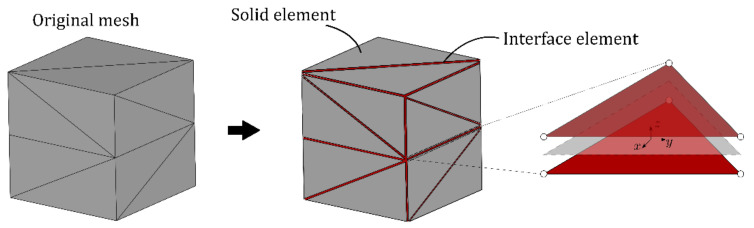
Insertion of zero-thickness interface elements into the finite element mesh.

This work focuses on the macroscopic response of the notched prismatic concrete specimens, describing concrete as a homogeneous material with effective material properties listed in Table 3. All material nonlinearities due to cracking are modeled by the cohesive-frictional zero-thickness interface elements, while the bulk of concrete is assumed to behave as a linear elastic material. Zero-thickness interface elements are inserted between the solid finite elements in the part of the domain where cracking is expected, as illustrated in Figure 5.

**Figure 6:**
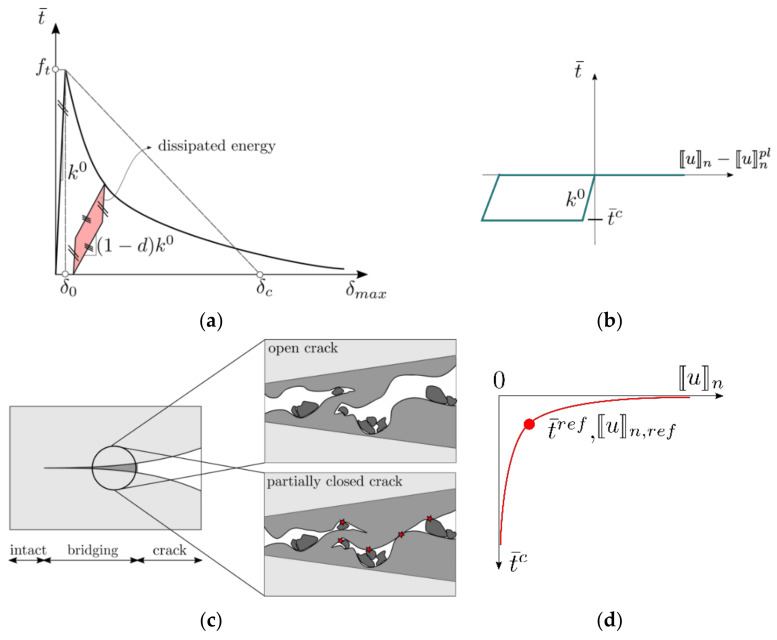
(**a**) Exponential softening relation used to model progressive stiffness and strength degradation due to cracking; (**b**) Schematic representation of the 1D plastic crack closure law; (**c**) Illustration of rough crack closure; contacts between asperities are marked with red stars; (**d**) hyperbolic relation between normal crack opening ${〚u〛}_{n}$ and the crack closure stress ${\bar{t}}^{c}$.

For non-cracked interface elements to remain closed, each interface element is equipped with high initial stiffness $k^{0}$. The stiffness and the residual strength degradation are governed by a softening traction-separation relation that postulates a relation between the equivalent traction $\bar{t}$ and the maximal equivalent opening $\delta_{\max}$. Here, $\delta_{\max}$ is a history variable that tracks the maximal historical value of the current equivalent opening (δ) so that the relation $f=\delta_{\max}-\delta \leq 0$ is always fulfilled. The value of equivalent opening δ is defined in terms of the crack opening displacement vector 〚u〛 as proposed in [36]:

(2) $$\delta =\sqrt{{〚u〛}_{n}^{2}+\frac{\beta^{2}}{\kappa^{2}}\left({〚u〛}_{t1}^{2}+{〚u〛}_{t2}^{2}\right)},$$

where ${〚u〛}_{n}$, ${〚u〛}_{t1}$ and ${〚u〛}_{t2}$ are the normal and the tangential (shear) components of 〚u〛, β is a scaling factor that accounts for the ratio of the shear and the tensile strength of concrete, and κ is a scaling factor that accounts for the ratio of the mode II ($G_{F,\mathrm{II}})$ and mode I ($G_{F,I}$) fracture energies as proposed by [36,37]. The softening relation is used for the concrete only. It is characterized by the following parameters: tensile strength $f_{t}$, fracture energy $G_{F,I}$, and the softening curve’s shape. A typical exponential t−δ softening relation, used in this work, is shown in Figure 6 and reads:

(3) $$\bar{t}=f_{t}e^{-2\frac{\delta_{\max}-\delta_{0}}{\delta_{c}-\delta_{0}}}.$$

$\delta_{0}=f_{t}/k^{0}$ and $\delta_{c}=2G_{F,I}/f_{t}+\delta_{0}$ are the derived quantities dependent on tensile strength $f_{t}$, initial stiffness $k^{0}$, and fracture energy $G_{F,I}$. The crack closure is modeled by a sub-model recovering the initial interface stiffness progressively as the microcracks close, which is explained in detail later in this section. The cohesive traction acting on the interface element is evaluated as follows:

(4) $$t=\frac{\bar{t}}{\delta_{\max}}k^{0}\left[\begin{array}{c} {〚u〛}_{n}\\ \frac{\beta^{2}}{\kappa }{〚u〛}_{t1}\\ \frac{\beta^{2}}{\kappa }{〚u〛}_{t2} \end{array}\right]+d\;t^{c}=\left(1-d\right)\;k^{0}\left[\begin{array}{c} {〚u〛}_{n}\\ \frac{\beta^{2}}{\kappa }{〚u〛}_{t1}\\ \frac{\beta^{2}}{\kappa }{〚u〛}_{t2} \end{array}\right]+d\;t^{c},$$

where d is the damage parameter, and the $t^{c}$ denotes the traction that arise due to progressive microcrack closure [38]. The surfaces of cracks are assumed to be rough, which leads to contacts between crack surfaces even before the cracks are completely closed, as illustrated in Figure 6c. The traction $t^{c}$ originating from these contacts is calculated according to a 1D plastic law in the direction normal to the crack surface, with two yield criteria: $f_{1}=t_{n}\leq 0$ and $f_{2}=-t_{n}+{\bar{t}}^{c}\;\leq 0$, where ${\bar{t}}^{c}$ is the “crack closure stress” [38]. Since for slightly open cracks, there is going to be many more contacts between the opposing surfaces than for very large opening, it is not realistic that crack closure stress remains constant. Therefore, we have adopted an empirical hyperbolic relation, illustrated in Figure 6d, that relates the current opening and the “crack closure stress” [27]:

(5) $${\bar{t}}^{c}=\frac{{\bar{t}}^{\mathrm{ref}}{〚u〛}_{n,\mathrm{ref}}}{{〚u〛}_{n}+b\;{〚u〛}_{n,\mathrm{ref}}},$$

where ${\bar{t}}^{\mathrm{ref}}$ and ${〚u〛}_{n,\mathrm{ref}}$ are fitting parameters and b is a small number compared to ${〚u〛}_{n,\mathrm{ref}}$ that enables avoiding division by zero in case of complete crack closure. The reference closure stress ${\bar{t}}^{\mathrm{ref}}$ is typically chosen to be equal to the splitting tensile strength. This plastic law results in evolution of plastic opening ${〚u〛}_{n}^{\mathrm{pl}}$, or also called internal sliding [39], that ensures satisfaction of two above mentioned yield criteria. Physically, this internal sliding causes dissipation of energy that manifests itself in the form of hysteresis in the load-displacement diagram.

### 5.2. Discrete Fiber Model

**Figure 7:**
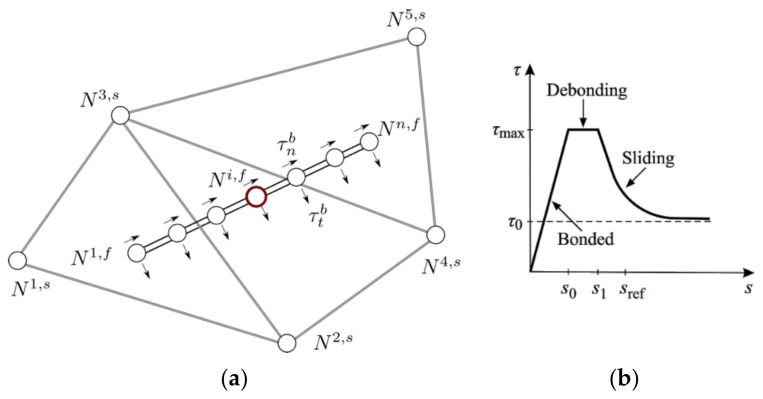
(**a**) Discretely modeled fiber using multiple Bernoulli beam elements. Each node *i* (marked with a red circle) of the fiber element *f* is tied to the corresponding solid element *s* through the bond element *b*. Bond elements provide resistance to axial sliding and lateral motion of fibers embedded in the solid matrix. (**b**) Illustration of three stages of the bond-slip law and meaning of bond parameters.

In this work, the short steel fibers are resolved explicitly, using the standard Bernoulli beam finite elements (e.g., [40]) embedded into solid finite elements that model the behavior of concrete, as shown in Figure 7a. To couple fibers and concrete, we utilize so-called bond elements. The role of these bond elements is to couple fiber and concrete domains by transferring forces between them and thus prevent their free relative motion. This coupling is achieved by applying a penalty constraint that imposes an additional relationship between the displacement of each fiber node and the displacement of the corresponding solid element at the same location. Assuming the configuration shown in Figure 7a, the bond-slip in the global coordinate system evaluated at the location of the node *i* can be calculated as:

(6) $$〚u〛=N_{u}u=\left[N^{2,s}\;\;\;N^{4,s}\;\;\;N^{3,s}\;\;\;-1\right]\left[\begin{array}{c} u_{2,s}\\ u_{4,s}\\ u_{3,s}\\ u_{i,f} \end{array}\right],$$

where 〚−〛 denotes the jump operator, $N^{k,s}$ (*k* = 2, 4, 3) is a block matrix containing shape function values corresponding to the node *k* evaluated at the location of the fiber node *i*, **1** is a unity matrix, and $u_{k,*}$ (* = s, f) is a vector of containing global degrees of freedom of the node *k* that belongs to either the solid element (s) or the fiber (f):

(7) $$N^{k,s}=\left[\begin{array}{ccc} N^{k} & 0 & 0\\ 0 & N^{k} & 0\\ 0 & 0 & N^{k} \end{array}\right]\;1=\left[\begin{array}{ccc} 1 & 0 & 0\\ 0 & 1 & 0\\ 0 & 0 & 1 \end{array}\right]\;u_{k,*}=\left[\begin{array}{c} u_{k,*}\\ v_{k,*}\\ w_{k,*} \end{array}\right].$$

The slip obtained in this way needs to be transformed into the local coordinate system of the fiber before the stresses, and consequently, internal forces can be calculated. The transformation is achieved by recourse to a rotation matrix in the following way:

(8) $$s=\left[\begin{array}{c} s_{n}\\ s_{t1}\\ s_{t2} \end{array}\right]=R〚u〛=\left[\begin{array}{ccc} n_{1} & n_{2} & n_{3}\\ t_{11} & t_{12} & t_{13}\\ t_{21} & t_{22} & t_{23} \end{array}\right]\left[\begin{array}{c} 〚u〛\\ 〚v〛\\ 〚w〛 \end{array}\right],$$

with n being the vector in the direction of the fiber, and $t_{1}$, and $t_{2}$ two vectors orthogonal to the fiber. The bond in the direction perpendicular to the fiber is considered always to remain intact, and the bond in the axial direction is assumed to follow the elastic-plastic bond slip relationship as illustrated in the Figure 7b, where the axial bond stress τ depends on the axial slip $s=s_{n}$ according to the following relation [41]:

(9) $$\tau \left(s\right)=\left\{\begin{array}{cc} K_{f}s & \;s\;\leq s_{0}\;\left(\mathrm{fully}\;\mathrm{bonded}\;\mathrm{stage}\right)\\ \tau_{\max} & ,\;s_{0}<s\;\leq s_{1}\;\left(\mathrm{debonding}\;\mathrm{stage}\right)\\ \tau_{0}+\left(\tau_{\max}-\tau_{0}\right)e^{\left(s_{1}-s\right)/s_{\mathrm{ref}}} & \;s>s_{1}\left(\mathrm{sliding}\;\mathrm{stage}\right) \end{array}\right.$$

where $K_{f}$ is the initial elastic stiffness of the bond, $\tau_{\max}$, $\tau_{0}$, $s_{0}$, $s_{1}$, and $s_{\mathrm{ref}}$ are the bond parameters that are calibrated based on the pullout experiments of the single straight fiber without hooks. Since this is an elastic-plastic law, the unloading stiffness is equal to $K_{f}$, and upon complete unloading, some residual plastic slip remains.

### 5.3. Finite Element Model of the Notched Fiber-Reinforced Prism Subjected to the Uniaxial Cyclic Load

**Figure 8:**
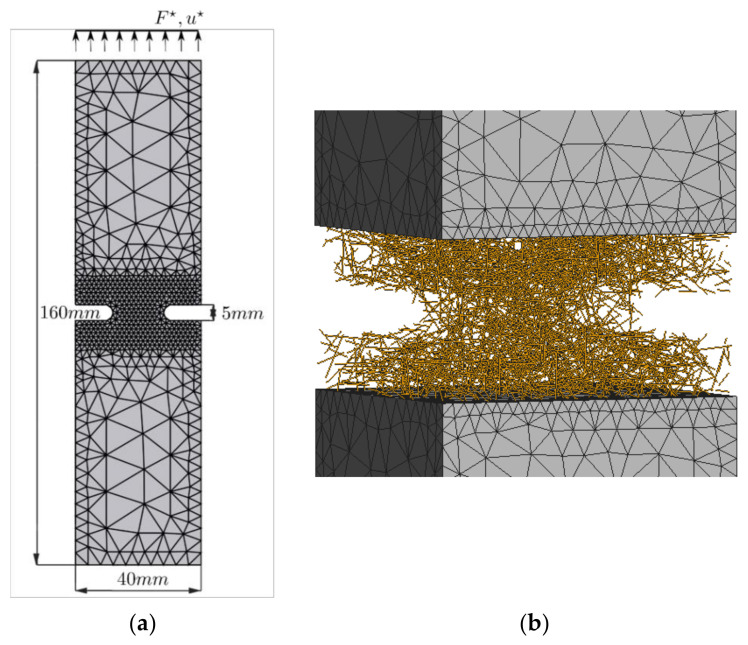
(**a**) The front view showing the dimensions of the notched prism model. Dimensions not shown in the figure: The depth of the notch is 10 mm, and the thickness of the specimen is 40 mm. Boundary conditions: motion of the bottom surface is precluded in all three spatial directions, the horizontal motion of the top surface is precluded, and the vertical displacement $(u^{*}$) of the top surface is prescribed according to the experimental program. (**b**) The side view of randomly distributed and oriented fibers in the central part of the notched prism specimen (solid and interface elements hidden).

A 3D finite element model of the notched prismatic specimen (front view of the mesh and dimensions shown in Figure 8a) was subjected to cyclic loading according to the experimental program described in detail in Section 4.2 and Section 4.3. Approximately 3500 fibers, randomly oriented according to isotropic distribution, corresponding to the 1% volume fraction (or ~78.5 kg/m^3^), were generated in the middle part of the specimen to model the fiber reinforcement, as shown in Figure 8b. These fibers and the bonds between fibers and concrete were modeled according to the description given in Section 5.2. As described before, in Section 4.3, a special loading/unloading scenario was devised for this experiment. To follow it precisely during the numerical simulation, in every time step, a crack mouth opening displacement (CMOD) was computed as an average of the openings of the left and the right notch. This value and the value of the total reaction force were used to determine the precise moments when the loading would stop, and the unloading of the specimen would begin. When the specimen would be almost totally unloaded, i.e., once the total reaction force reached a small value of approximately 0.1kN, loading would start again until the next unloading point is reached.

## 6. Results and Discussion

### 6.1. Pull-Out Tests

**Figure 9:**
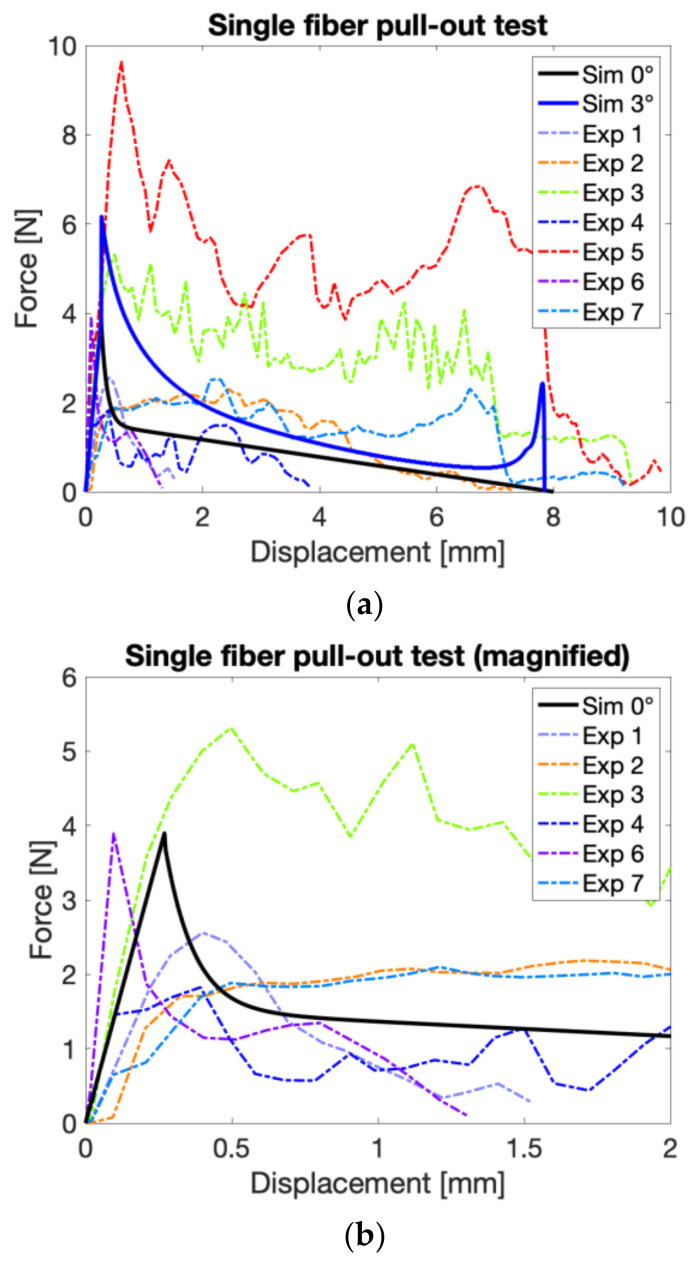
(**a**) Experimentally (dashed lines) and numerically (solid lines) obtained force versus slip curves from pull-out tests of individual fibers from the mortar matrix (0° indicates that fibers are parallel to the normal to the surface). It can be noticed that in several cases (Exp. 3, 5, 7), a sharp increase in the force is observed near the end of the pull-out experiment. This increase in pull-out could be replicated by the analytical pullout model when a slight inclination (Sim. 3°) was introduced. These tests are used to calibrate the bond-slip model (Table 5). (**b**) The magnified view shows the numerical fit to the experimental data on the smaller displacement range. Experiment 5 was not plotted because of the significant deviation in the peak load.

Pull-out tests of single fibers perpendicular to the free (crack) surface were used to investigate the interfacial bond strength and to calibrate the bond-slip law used in bond elements that tie discrete fibers and solid elements. The results of fiber pull-out tests (experimental and numerical) are shown in Figure 9a and the magnified view that shows the numerical fit to the experimental data on the smaller displacement range is shown in Figure 9b. The procedure used for the identification of bond parameters using a semi-analytical pullout model minimizes the deviation of the numerical response to the experimentally measured data, and it is described in detail in [42,43]. The parameters derived from this pull-out test are listed in Table 5. During the calibration procedure, we have assumed that the parameters s_0_ and s_1_ were equal. The consequence of this is that the plateau that occurs after yielding in Figure 7b vanishes. This choice is justified, as no plateau is noticeable in the experimental measurements, and also, the model is consequently simplified. Further, it should be noted that there is a relatively large scatter of the experimentally obtained results, which is typical for single fiber pullout tests, especially with very small fibers as used here. Therefore, we attempted to obtain the parameters that fit the average behavior of all pullout tests, assuming that this is valid when many fibers are pulled out simultaneously, such as while cracking during the above-described tensile test on the notched prismatic specimen.

It can be noticed that in several cases (Exp. 3, 5, 7), a sharp increase in the force is observed near the end of the pull-out experiment. This increase in pull-out could be replicated by the analytical pullout model when a slight inclination (Sim. 3°) was introduced.

### 6.2. Static and Cyclic Tensile Tests

**Figure 10:**
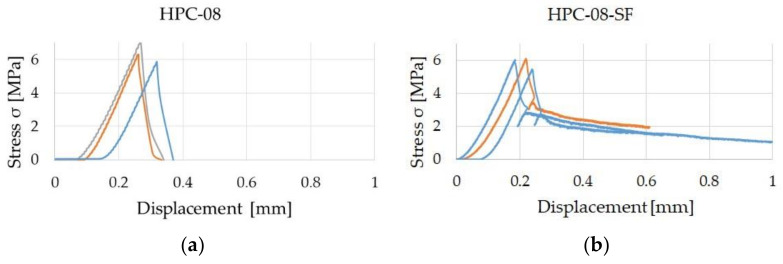
Stress-displacement diagrams. (**a**) HPC-08; (**b**) HPC-08-SF.

The stress-displacement curves from the static tensile tests of HPC-08 and HPC-08-SF are shown in Figure 10. The average maximum tensile strength is *f*_ct,max_ = 6.4 MPa for HPC-08 and *f*_ct,max_ = 5.9 MPa for HPC-08-SF. A change in the tensile strength due to the addition of steel microfibers could not be determined; the slight deviation between HPC-08 and HPC-08-SF can be attributed to the usual scatter of results in direct static tensile tests and possibly a slight influence of the fibers at primary resistance to cracking.

**Figure 11:**
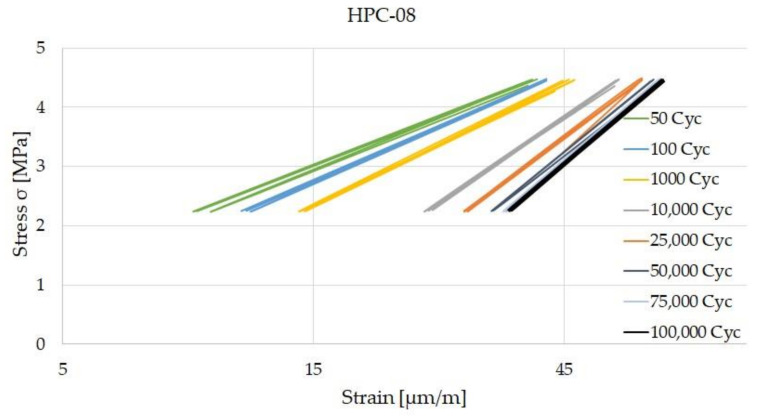
Stress-strain diagram of HPC-08 after 50 up to 100,000 Cycles.

**Figure 12:**
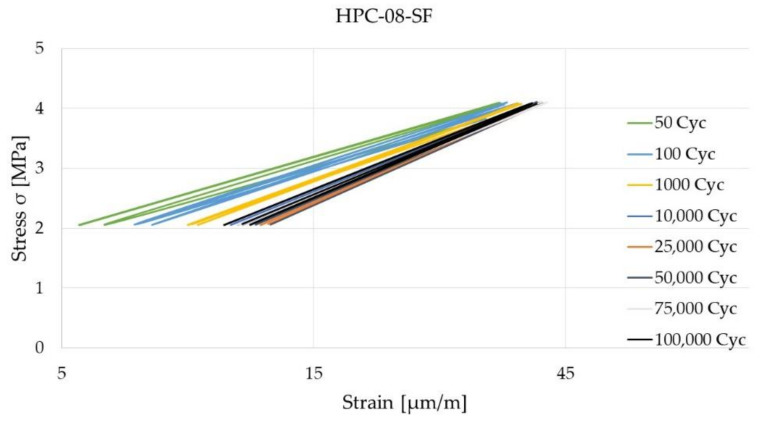
Stress-strain diagram of HPC-08-SF after 50 up to 100,000 Cycles.

Figure 11 and Figure 12 show the hysteresis after selected load cycles of HPC-08 and HPC-08-SF. HPC-08 shows a steady increase in strain until it reaches 100,000 load cycles. The maximum strain is around 70 µm/m. For the steel fiber reinforced high-strength concrete (HPC-08-SF), the strain stagnates from approximately 10,000 load cycles and does not go beyond 40 µm/m. That means that the rate of strain increase was lower for HPC-08-SF.

**Figure 13:**
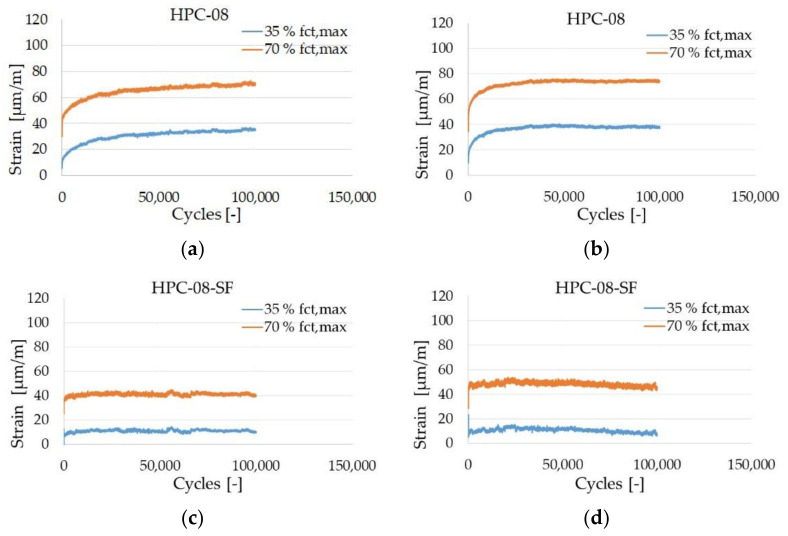
Examples of strain-cycle (S-N) diagrams of: (**a**,**b**) HPC-08; (**c**,**d**) HPC-08-SF up to 100,000 Cycles.

Systematic investigations of high-strength concrete with and without high-strength steel fibers subjected to static and cyclic tensile loadings were conducted to understand the influence of high-strength steel microfibers with particular regard to microcracking. Another aim of the study was to gain knowledge about the starting point of microcracks. The comparison of the strains of HPC-08 and HPC-08-SF is shown in Figure 13a–d. The strain in the vertical direction was continuously recorded using strain gages. It is shown that the strain of HPC-08 grew faster and showed a more significant increase in phase I of damage evolution. Comparatively, the strain of HPC-08-SF only grew marginally in phase I, and in phase II there was no noticeable increase in strain. HPC-08 shows a steady increase in strain in phase II of damage evolution. Both (HPC-08 and HPC-08-SF) show no transition to phase III of the damage evolution due to the limited number of load cycles.

**Figure 14:**
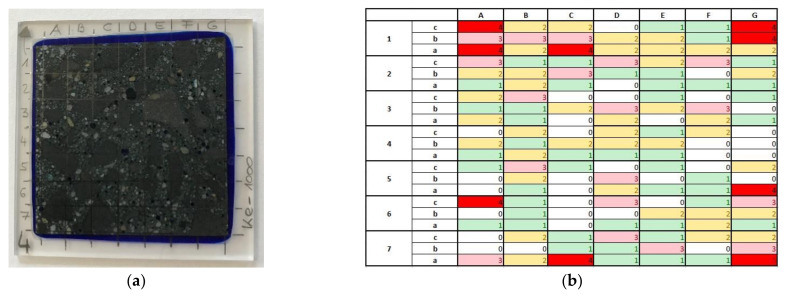
(**a**) Example of a thick section with examination field of 35 × 35 mm^2^ and a raster of 7 × 7 cells and (**b**) example of tabular examination of crack amount: white = no cracks; green = one crack; yellow = 2 cracks; pink = 3 cracks; red = more than 3 cracks.

Thick sections cut vertically (see Figure 14a) were taken from one intact test specimen and each of one test specimen after 1, 100, 1000 and 100,000 load cycles from HPC-08 and HPC-08-SF to characterize the microcracking of concrete in terms of the amount, length, width, and position of microcracks.

The microscopic investigations showed that when a specimen was subjected to cyclic loading, new microcracks would predominantly form rather than the existing ones grow. The HPC-08-SF specimen showed more microcracks after 100,000 load cycles, but these were smaller in size, and when compared to the plain high-strength concrete, the crack pattern was finer. Thus, the total crack area in the HPC-08-SF specimen was lower, as can be seen from Table 6 and Table 7. Around 66% of the microcracks have initiated in the so-called interfacial transition zone (ITZ) between the aggregate and the hardened cement stone in both HPC-08 and HPC-08-SF, indicating that the ITZ is the weakest zone in the case of high-performance concretes. The remaining microcracks developed in the hardened cement stone and through aggregates. If the fibers in the matrix have sufficient tensile strength and stiffness, they can act as reinforcement delaying macrocrack formation and limiting the opening. If the cracks are not too wide and the fibers are adequately anchored in the matrix, the bridging of the crack faces is possible, transmitting tensile forces across the crack. The transmitting of tensile forces between the crack faces leads to a finely distributed crack pattern with smaller crack spacings and crack widths as a result of cyclic loading.

### 6.3. Crack Opening Tests

**Figure 15:**
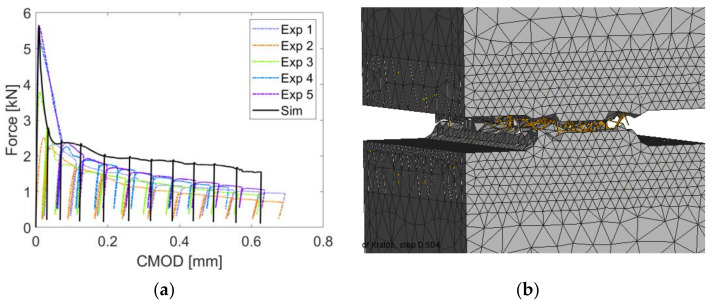
(**a**) Experimentally and numerically obtained force versus crack mouth opening displacement (CMOD) curves; (**b**) Side view of the numerically simulated fiber reinforced notched prismatic specimen.

**Figure 16:**
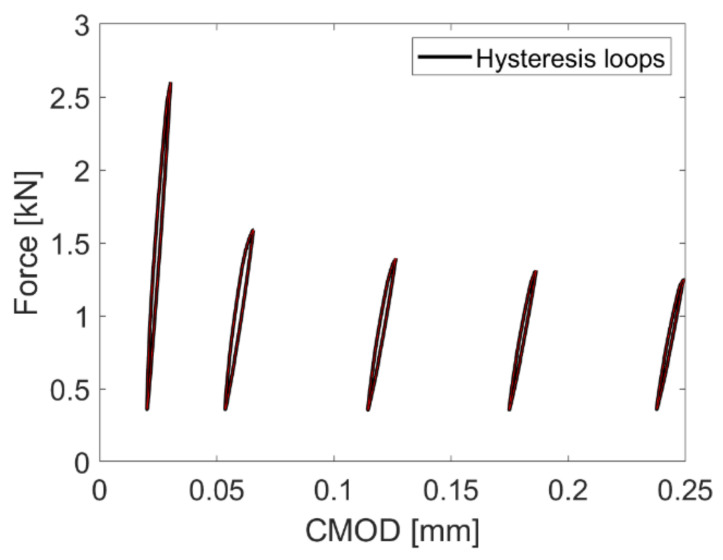
Extracted first five hysteresis loops due to unloading-reloading cycles in the Exp. 3.

This section investigates experimentally and numerically the post-cracking behavior of notched prismatic specimens subjected to the loading program described in Section 4.3. Five specimens, denoted as Exp. 1 to Exp. 5, were subjected to cyclic loading in the experimental campaign. The measured force versus crack mouth opening displacement (CMOD) diagrams are presented with dashed lines in Figure 15a, and with a thick black line, the response of numerically simulated fiber-reinforced notched prismatic specimen is shown. In Figure 15b, the simulated cracked numerical model with clearly shown discretely modeled short steel fibers is shown. The numerical model captures well the overall response of the short steel fiber-reinforced specimen under cyclic loading. It can be noted that the addition of fibers did not strongly influence the tensile strength and that the maximal load obtained numerically (5.63 kN) corresponds to the nominal tensile strength of 7.03 MPa, whereas the tensile strength of plain concrete was chosen as 6.4 MPa in the simulation. Due to the large scatter of experimental results (especially experiments 1 and 2), the nominal tensile strength of the fiber-reinforced concrete was lower than the one of the plain concrete and amounted to 5.77 MPa. It can be noted in the response diagrams in Figure 15a that the presence of fibers had a strong influence on the residual strength and the unloading behavior. The residual strength is slightly overestimated in numerical simulations in later stages of the experiment. In experiments, the unloading stiffness after cracking is slightly lower than the initial (elastic) stiffness, and as cycling progresses, the stiffness reduction in each subsequent cycle is relatively small. Further, the unloading-reloading cycles generate very small hysteresis loops (see Figure 16, where the first five loops of experiment 3 are shown). Both the fact that the unloading stiffness remains stable and that hysteresis loops are small indicates that the residual strength comes primarily from fibers carrying the load and that the dissipation manifested in the form of a hysteresis stems primarily from the frictional sliding of fibers in fiber channels during unloading and reloading.

## 7. Conclusions

Experimental and numerical studies were carried out on high-strength concrete with and without short steel fibers. The described test procedure led to the following conclusions regarding the specific stress levels and stress amplitude:

1. The cyclic tests showed that the rate of strain increase was lower for HPSFRC specimens and that the strain stagnated after around 10,000 cycles, which was not the case with HPC specimens.
2. The microscopic analysis shows that the number of cracks increased continuously throughout the cyclic tests. In the case of high-strength concrete without short steel fibers (HPC-08), wider and longer cracks and thus a larger total crack area was found. In the HPC-08-SF, overall, more cracks were found, but these were smaller and shorter, i.e., more finely distributed so that the total crack area was smaller than in case of HPC-08.
3. More than 66% of the microcracks run or start in the contact zone between the aggregate and the hardened cement stone (ITZ).
4. The controlled crack opening tests were conducted to investigate the post-cracking behavior of short steel fiber reinforced concrete (HPC-08). The experiments have shown, and the numerical simulations have confirmed that the addition of short steel fibers had a minimal influence on the tensile strength, but the fibers significantly improved the ductility. The unloading stiffness after cracking is slightly lower than the initial (elastic) stiffness; however, it remains stable during the rest of the experiment, even under large crack openings. During unloading/reloading cycles, only a small hysteresis loop is formed, which, together with relatively stable unloading stiffness, indicated that the fibers carried the majority of the load. The dissipation due to frictional sliding of fibers manifested itself in the formation of the small hysteresis on the response diagram.

## Figures and Tables

**Table 1:** Compositions of high-performance mortar (HPC-02) and concrete with (HPC-08-SF) and without fibers (HPC-08).

Components		HPC-08	HPC-08-SF	HPC-02
Cement	[kg/m^3^]	500	500	646
Sand 0/0.5	[kg/m^3^]	75	75	150
Sand 0/2	[kg/m^3^]	850	850	1333
Basalt 2/5	[kg/m^3^]	350	350	-
Basalt 5/8	[kg/m^3^]	570	570	-
Superplasticizer	[kg/m^3^]	5.0	6.25	4.85
Stabilizer	[kg/m^3^]	2.85	3.55	2.76
Water	[kg/m^3^]	176	176	227.4
Steel fibers	[kg/m^3^]	-	78.5	-
*w*/*c*	[-]	0.35	0.35	0.35

**Table 2:** Properties of the high-strength short steel fibers.

Material	Description	Brand	Length (mm)	Diameter (mm)	Tensile Strength (MPa)
Steel	Short fibers	Dramix OL 6/0.16	6	0.160	2600
Steel	Long fiber	-	250	0.155	2345

**Table 3:** Properties of the high-performance concrete.

Material Property	Value
Tensile strength (*f*_t_) [MPa]	6.4 *
Compressive strength [MPa]	109.0 *
Ratio of shear and tensile strength (*β* = *f*_s_/*f*_t_) [-]	3.5 **
Fracture energy in mode I (*G*_F, I_) [J/m^2^]	100.0 *
Ratio of fracture energies in mode II and mode I (*κ* = *G*_F, II_/*G*_F, I_) [-]	10 **
Young’s modulus [MPa]	43,224.4 *
Poisson’s ratio [-]	0.2 *
Reference closure stress (${\bar{t}}^{\mathrm{ref}}$) [MPa]	6.4 ***
Reference closure opening (${〚u〛}_{n,\mathrm{ref}}$) [mm]	0.4 ***

* Own measurements. ** [36]. *** Calibrated model parameters.

**Table 4:** Applied loads.

Composition		HPC-08	HPC-08-SF
Lower Load 35% *f*_ct,max_	[kN]	1.79	1.64
Upper Load 70% *f*_ct,max_	[kN]	3.58	3.28

**Table 5:** Properties of the fiber-concrete bond evaluated from single fiber pullout tests.

Fiber Bond Properties	Value
*τ*_max_ [MPa]	1.0
*τ*_0_ [MPa]	0.4
*s*_0_ [mm]	0.001
*s*_1_ [mm]	0.001
*s*_ref_ [mm]	0.1

**Table 6:** Microscopic analysis of HPC-08.

Composition		HPC-08				
Cycles		0	1	100	1000	100,000
Number of cracks	[-]	136	161	177	216	229
Mean crack width	[µm]	6.38	6.48	6.59	6.69	8.39
Mean crack length	[µm]	467	612	652	649	835
Mean crack area	[µm^2^]	3050	4030	4490	4510	7200
Total crack area (35 × 35 mm^2^)	[µm^2^]	414,484	648,357	795,573	974,455	1,649,470

**Table 7:** Microscopic analysis of HPC-08-SF.

Composition		HPC-08-SF				
Cycles		0	1	100	1000	100,000
Number of cracks	[-]	145	182	218	244	302
Mean crack width	[µm]	5.18	4.69	4.55	4.20	5.39
Mean crack length	[µm]	421	457	447	540	511
Mean crack area	[µm^2^]	2230	2240	2070	2350	2870
Total crack area (35 × 35 mm^2^)	[µm^2^]	322,677	407,381	451,006	573,081	866,674

## Data Availability

Not applicable.
